# Tooth Loss, Cognitive Impairment and Fall Risk: A Cross-Sectional Study of Older Adults in Rural Thailand

**DOI:** 10.3390/ijerph192316015

**Published:** 2022-11-30

**Authors:** Niruwan Turnbull, Pichayasuda Cherdsakul, Sutin Chanaboon, David Hughes, Kukiat Tudpor

**Affiliations:** 1 Faculty of Public Health, Mahasarakham University, Maha Sarakham 44150, Thailand; 2 Public Health and Environmental Policy in Southeast Asia Research Unit (PHEP-SEA), Mahasarakham University, Maha Sarakham 44150, Thailand; 3 Sirindhorn College of Public Health Khon Kaen, Khon Kaen 40000, Thailand; 4 Faculty of Medicine, Health & Life Science, Swansea University, Swansea SA2 8PP, UK

**Keywords:** tooth loss, older persons, cognitive impairment, fall risk, rural area

## Abstract

Tooth loss is associated with both cognitive impairment and fall risk. However, the relationships between these variables are complex and bidirectional. Observed associations have been reported in separate studies but data on rural-dwelling older adults remain sparse. This cross-sectional study investigated socioeconomic and dental factors affecting cognitive functions, and the association between tooth loss, cognitive functioning, and fall risk. Two hundred and thirty-one rural-dwelling older adults (60–74 years old) were recruited from a single Dental Service Unit. Cognitive function and fall risk were assessed with the Mini-Mental State Examination and the Morse Fall Scale, respectively. Oral examinations were performed by a dentist using the Community Periodontal Index of Treatment Needs form. 38.1%, had >16 tooth loss. Socioeconomic data and health status were obtained from a questionnaire and interviews. Age, Activities of Daily Living (ADL) score, and the number of teeth lost was significantly associated with impaired cognitive function. Chi-square analysis showed that cognitive function was also associated with fall risk. Past research suggests that much cognitive impairment and fall risk is induced by tooth loss. Service planners need to be aware of the complex bidirectional relationships between these variables and give higher priority to dental services that can improve the general health status and social functioning of older rural adults.

## 1. Introduction

Poor oral health, cognitive impairment, and fall risk are interrelated in complex ways. Chen and co-workers proposed that cognitive impairment was a cause of dental caries due in part to restricted activities of daily living and poor dental self-care [1], and cognitively impaired older adults have been shown to be at greater risk of falls [2], which may result in dental trauma. At the same time, there is accumulating evidence that tooth loss can itself cause cognitive impairment and increase fall risk. A systematic review and meta-analysis of 21 observational studies showed that tooth loss was a risk factor for dementia [3]. More recently, a systematic review by Dibello and colleagues highlighted a significant role of poor oral hygiene on age-related cognitive decline and frailty [4]. Luo and colleagues found that older adults with 16 or more teeth lost had increased levels of cognitive impairment and note that there are biologically plausible mechanisms via which tooth loss can cause impairment [5]. They cite past studies that show *inter alia* that periodontal pathogens can produce chronic inflammation and neuropathology, that periodontal disease raises the risk of cerebrovascular disease, that oral Gram-negative bacteria may reach the brain via transient bacteraemia, and that mercury released from dental amalgam can affect brain function. Minn and associates similarly argue that periodontal disease is a predictor of cognitive impairment, citing research that links tooth loss to subclinical vascular brain damage, white matter degeneration and micro bleeds, as well as the detrimental effects of poor nutrition [6].

Falls in older people are a global public health issue with an annual rate ranging from 4% to 35% affected being reported [7]. Recently, Monachan and colleagues reported that older persons living in semi-urban areas with cognitive impairment had approximately four times the risk of falls compared with those with normal cognitive function [2]. However, the relationship between cognitive functioning and fall risk among older persons living in rural areas with limited education and low incomes remains an under-researched topic.

Thailand’s universal coverage healthcare system is divided into three programs—a civil servant medical benefit scheme (CSMBS) covering 7% of the population, a social security scheme (SSS) for formal-sector private employees covering 15%, and a universal coverage scheme (UCS) for all other Thai citizens that covers about 76% of the population [8]. According to a recent study by Srinarupat and colleagues, low-income Thai people living in rural areas have a higher prevalence of gingivitis and periodontitis, but face the problem that the service charges for endodontic treatments and surgical removal of posterior teeth are not covered by the UCS [9]. Periodontitis is the major cause of tooth loss and affects masticatory functions and quality of life [10]. Additionally, poor oral health and periodontitis are associated with several extra-oral diseases such as diabetes mellitus, cardiovascular disease, rheumatoid arthritis, and ocular disease [11]. With the foregoing in mind, this present study aimed to investigate how far socioeconomic and dental factors were associated with cognitive functioning, as well as the association between cognitive functioning and fall risk in older rural-dwelling adults. We hypothesized that the combined effects of socioeconomic and dental factors predicted cognitive functioning in this population group.

## 2. Materials and Methods

### 2.1. Research Design

The participants in this cross-sectional study were selected from patients treated at the Dental Service Unit in Chik Thoeng subdistrict, Tan Sum district, Ubon Ratchathani province, Thailand. The protocol followed the STROBE checklist (Supplementary Material Table S1) for cross-sectional studies. Four hundred and thirty-one rural-dwelling adults aged from 60 to 74 years were screened to see if they met the inclusion criteria. The criteria were being residents of Chik Thoeng subdistrict, aged ≥ 60, being able to read and write in Thai, consenting to participate in the research, and satisfactory cognitive function. The potential participants were resident in nine rural villages in the subdistrict, and the team decided to preserve the geographical spread of patients by stratified sampling of roughly equal numbers from each village. It had been decided to exclude patients with dementia because of likely inability to complete questionnaires and interviews. Cognitive function was assessed using the Mini-Mental State Examination (MMSE) assessment tool. The license for using the MMSE, 2nd edition, was purchased from https://www.parinc.com/products/pkey/237 accessed on 11 November 2022 with Web Order Number 11193220 and Item Number 10255-EM. MMSE-Thai 2002 scores range from 0–30. Based on the educational backgrounds of the participants, the cut-off scores were modified. For older persons who were illiterate or had not finished elementary school, the cutoff score was 14, for elementary education it was 17, and for those with education beyond primary level it was 22 [12,13,14]. 231 patients in the latter two categories were included in the main phase of the research. The study design was approved by the Ethical Review Committee for Human Research, Mahasarakham University (Study No. 019/2563). Informed consent forms were completed by all participants. A required sample size (*n* = 231) was calculated using Krejcie and Morgan’s formula [15] as follows.

$$n=\frac{X^{2}NP\left(1-P\right)}{e^{2}\left(N-1\right)+X^{2}P\left(1-P\right)}$$

*n* = required sample size.

*X*^2^ = the table value of Chi-square for 1 degree of freedom at the desired confidence level (3.841).

*N* = the population size.

*p* = the population proportion (assumed to be 0.5 since this would provide the maximum sample size).

*e* = the degree of accuracy expressed as a proportion (0.05).

**Figure 1:**
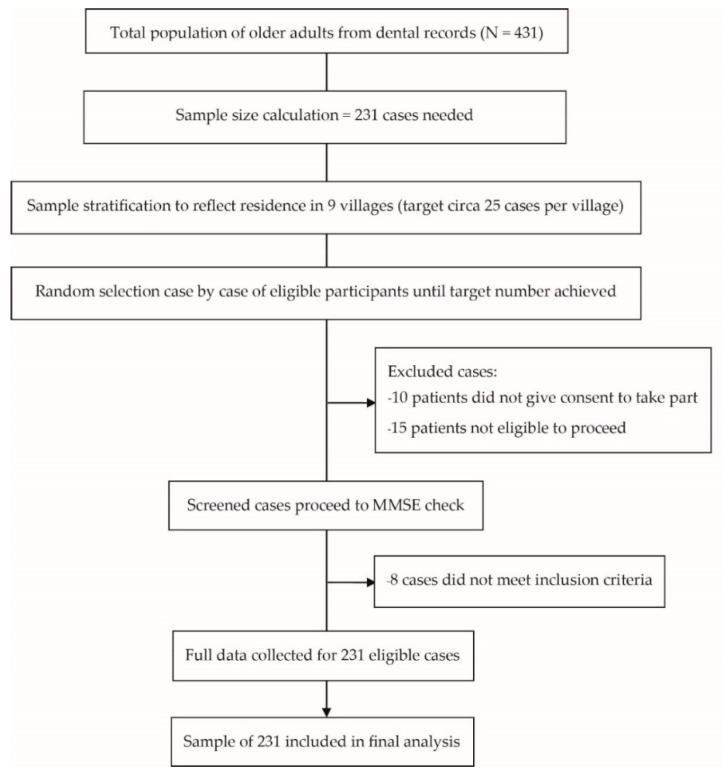
Sample collection flow chart.

Stratified random sampling to recruit roughly equal numbers from each of the nine villages was used to select 231 participants from the 431 rural-dwelling adults. Recruitment of eligible patients was repeated in each village in turn until the required number of participants was reached (Figure 1). Socioeconomic data (age, sex, education levels, family status, congenital disease, body mass index [BMI], activities of daily living [ADLs], and betel chewing) were obtained from a questionnaire, which comprised both closed and open-ended questions. The investigators involved in the assessment were blinded to the random sampling process. The ADLs were evaluated using Barthel’s ADL index with a Cronbach’s alpha reliability coefficient of 0.90 [16]. The evaluation form had ten items on the ADL checklist on bathing, dressing, bowel and bladder care, feeding, grooming, climbing stairs, ambulation, transfer, and toilet use. The summed ADL scores ranged from 0–20 and were divided into two groups—dependent (<12) and independent (≥12). Some older adults in Thailand chew betel nuts, which is associated with dental caries and periodontitis [17]. Therefore, data on betel chewing were also collected.

Data on health status (chronic disease, blood pressure, blood glucose levels, et cetera) were obtained directly from face-to-face interviews and supplemented by records data from the Java Health Center Information System (JHCIS), Ministry of Public Health. Where there were discrepancies, information was completed after consulting local district hospital staff. Fall risk was measured using the Morse Fall Scale (MFS). The MFS consists of 6 items: history of falling (scored no = 0, yes = 35), ambulatory aid (bed rest/nurse assist = 0, cane/crutches/walker = 15, special furniture/appliances = 30), secondary diagnosis (no = 0, yes = 15), gait (normal/immobile/bed rest = 0, weak = 10, impaired = 20), intravenous (IV) or heparin lock (no = 0, yes = 20), and mental status (oriented to own ability = 0, forgets limitations = 15). In the present study, the participants with MFS scores <45 and ≥45 were classified as low risk and high risk of fall, respectively. Oral examination of respondents was performed by a dentist. The Community Periodontal Index of Treatment Needs (CPITN) form was used to assess periodontal status [18]. In line with World Health Organization criteria, the Decayed, Missing and Filled Tooth (DMFT) Index was employed and a detection record form was used to record the number of decayed, missing, and filled teeth [19]. Since Luo and co-workers reported that missing >16 teeth was associated with cognitive impairment, we set this cutoff point for our data analysis [5].

### 2.2. Statistical Analysis

The data were analyzed using the Statistical Package for Social Sciences (SPSS version 18, SPSS, Inc., Chicago, IL, USA). Descriptive statistics were assembled using average values, standard deviation (SD), and percentage distribution. The Chi-square (χ^2^) test and Fisher’s exact test were used to investigate the relationship between categorical variables. Binary logistic regression analysis was used to analyze predictive values of socioeconomic characteristics that might be associated with cognitive impairment. Prerequisites for binary logistic regression were checked for multicollinearity amongst independent variables and independence between observations. Stepwise binary logistic regression was used because this excludes variables that do not contribute to explaining differences in the dependent variable. The independent variables were age [<70 vs. ≥70 years old], ADLs [<12 versus ≥12] [20], and the number of teeth lost [≤16 vs. >16] [5], and were analyzed using predicted binary variables (normal vs. impaired cognitive function). The results from the binary logistic regression are expressed as odds ratios. Odds ratios >1 indicate that the event is more likely to occur as the value of the predictor increases. Conversely, odds ratios <1 indicate that the event is less likely to occur as the predictor value increases. The statistical significance level was set at *p* < 0.05.

## 3. Results

### 3.1. Characteristics of General and Dental Status of Older Adults

The general and dental characteristics of 231 participants are shown in Table 1. 54.5% were male, 66.2% aged under 70 years old, 69.7% married, 17.7% educated in secondary school or higher, 69.3% had normal BMI, 93.9% had ADL scores indicating independent functioning, and 64.5% had no congenital disease. Regarding dental status, 21.3% chewed betel nuts, 38.1% had >16 teeth lost, 21.2% had decay in ≥1 teeth, 10.8% had ≥1 dental fillings, 14.7% had <7 pairs of opposing post-canine teeth, and 51.1% had calculus (dental tartar).

### 3.2. Factors Predicting Cognitive Impairment in Older Adults

The association of the various participant characteristics with the level of cognitive functioning was investigated using Fisher’s exact test or the Chi-square test. Only the factors associated with cognitive impairment were further analyzed via binary logistic regression to determine the predictive power of the independent variables. Results showed that age, ADLs, and the number of teeth lost were significantly associated with the likelihood that patients suffered an impaired cognitive function at *p* < 0.05 (Table 2). Participants aged ≥70, with ADLs <12, and >16 teeth lost were, respectively, 2.64, 4.89, and 4.29 times more likely to have an impaired cognitive function than those aged <70, with ADLs ≥12, and ≤16, teeth lost.

### 3.3. Association between Cognitive Function and Fall Risk

Association between cognitive functioning and fall risk was analyzed using the χ^2^ test. Results showed that cognitive functioning was significantly associated with fall risk at *p* < 0.001 (Table 3).

## 4. Discussion

This study found that number of teeth lost is associated with cognitive impairment and cognitive impairment is related to fall risk in older rural-dwelling adults. Our conclusion is based on the following findings: (1) number of teeth lost is a predicting factor for cognitive impairment and (2) cognitive impairment is associated with fall risk.

The relationship between tooth loss, cognitive impairment and fall risk is complex and bidirectional. In Thailand a large number of older adults are affected by high rates of tooth loss [20]. To date, the links between tooth loss, cognitive impairment, and fall risk have been separately reported for different population groups, and more evidence is needed before the overall picture becomes clear. The more intuitively plausible direction of causality is that adults with cognitive impairment and increased fall risk are more likely to lose teeth because of restricted ADLs and poor self-care [21]. However, as noted earlier, many researchers suggest that tooth loss itself predicts impaired cognitive function and fall risk. Periodontal disease has been linked to changes in the cerebrum, cerebral cortex and white matter functions [6,22]. Akashi and colleagues’ narrative review suggests that odontogenic infection can lead to intracranial infection, particularly temporal lobe infection [23]. Yang and associates report that bacteria diffused from odontogenic foci to frontoparietal areas [24], areas responsible for attention control [25]. The medial area of the temporal lobe controls memory function as evidenced by medial temporal lobe atrophy in elderly individuals with memory impairment [26]. Another possible mechanism affecting brain function is bacteriological infection via neuronal pathways, such as the cranial nerve [27]. Lastly, a systematic review by Wang and colleagues concluded that mastication positively affected cerebral blood flow [28]. Tooth loss reduces masticating ability and this may result in reduced cerebral blood flow that subsequently impairs cognitive functions and the motor skills necessary for standing and walking balance. Both static and dynamic balance are well-known protective factors against falls in older persons [29].

Plozer and colleagues suggest that investigation of pathways by which tooth loss-related morbidity and mortality is mediated will increase the perceived value of dental treatment for general health [30]. Tooth loss without use of dentures is said to be an independent predictor of incident falls in older adults without cognitive impairment [31]. These authors propose that people with tooth loss are susceptible to depression, which is a risk factor for falls [32]. In many cases depression is associated with cognitive problems [33], which makes it difficult to isolate tooth loss from cognitive impairment, supporting our finding of an association between cognitive impairment and high risk of falls in older persons.

Access to dental treatment is likely to become more difficult for rural populations in a period of economic turbulence and constrained public expenditure, especially in developing countries such as Thailand [34]. Our participants were older rural-dwellers who mostly live at a distance from medical services. Most only received basic dental care such as scaling and tooth extraction due to the limited coverage provided by the UCS [9]. We have provided further evidence of the association between number of teeth lost, cognitive impairment and fall risk. If the relationships are indeed bidirectional then tooth loss has an impact on the older rural population that goes far beyond matters like appearance and eating to affect general health status and social and cognitive functioning. Service planners need to take account of these wide-ranging impacts to give higher priority to dental services as a determinate of general health status, and make corresponding improvements to patterns of service delivery.

The nature of our cross-sectional study means we must be cautious about our conclusions. While we found an association between number of teeth lost and degree of cognitive deficit (magnitude), we did not collect data over time that would illuminate whether deficits increase as teeth are lost (temporal change). While desirable a “before and after” design was not feasible in the period available for this study. For this reason, we cannot claim evidence of a definitive causal link between tooth loss and the two other variables; our finding that number of teeth lost is associated with degree of cognitive deficit is consistent with that hypothesis but further research is needed.

Another limitation of the study was the lack of data on determinants of physical strength such as lower limb muscle power and postural balance, which have been shown to reduce fall risk in older adults [35]. These factors may be associated with age-related muscle degeneration and a decline in physical activity [36]. Tooth loss-induced malnutrition and sequential muscle protein wasting should also be considered for further study [37]. Finally, the small scale of our study in a single location means that we must be cautious about the generalizability of the results to other populations; our findings contribute to a jigsaw puzzle picture that will need to be assembled from a number of studies.

## 5. Conclusions

In conclusion, the present study found an association between number of teeth lost, cognitive impairment and fall risk among rural-dwelling older adults. However, further research using larger study populations needs to be performed to verify whether this is a major public health problem. If our findings are supported, then health planners need to develop strategies for improving service delivery for this population group.

## Figures and Tables

**Table 1:** Characteristics and dental status of participants (*N* = 231).

Variable	*n*	%
**Gender**		
Female	105	45.5
Male	126	54.5
**Age**		
60–69	153	66.2
≥70	78	33.8
**Marital status**		
Married	161	69.7
Single/divorced/Widowed/Separated	70	30.3
**Educational level**		
No schooling	87	37.7
Primary school	41	17.7
Higher than primary school	103	44.6
**BMI (kg/m^2^)**		
Normal (≥25 to≤35)	71	30.7
Health risk (<25 or >35)	160	69.3
**ADLs**		
<12	14	6.1
≥12	217	93.9
**Congenital disease**		
No	149	64.5
Yes	82	35.5
**Betel nut chewing**		
Never	181	78.7
Been chewing betel nuts	49	21.3
**Number of teeth lost**		
≤16 teeth	143	61.9
>16 teeth	88	38.1
**Number of decayed teeth**		
None	182	78.8
≥1 tooth	49	21.2
**Number of dental fillings**		
None	206	89.2
≥1 tooth	25	10.8
**Number of opposing post-canine tooth pairs**		
≥7	197	85.3
<7	34	14.7
**Calculus**		
Not present	113	48.9
Present	118	51.1

**Table 2:** Factors associated with level of cognitive functioning among participants.

Factors	Cognitive Function Level				Crude OR (95% CI)	Adjusted OR (95% CI)	*p*-Value
	Normal (*n* = 149)		Impaired (*n* = 82)				
	*n*	%	*n*	%			
**Age**							
60–69	110	71.9	43	28.1	Ref	Ref	Ref
≥70	39	50.0	39	50.0	0.39 (0.22–0.68)	2.64 (1.25–5.57)	0.010
**ADLs**							
≥12	145	66.8	72	33.2	Ref	Ref	Ref
<12	4	28.6	10	71.4	5.03 (1.52–16.60)	4.89 (1.17–20.40)	0.029
**Number of teeth lost**							
≤16 teeth	113	79.0	30	21.0	Ref	Ref	Ref
>16 teeth	36	40.9	52	59.1	5.44 (3.03–9.76)	4.29 (2.06–8.91)	<0.001

Note: Ref indicates a value chosen as the reference value to which odds ratios (OR) were compared.

**Table 3:** Cognitive function and fall risk.

Cognitive Function	Fall Risk		Total	95% CI	χ^2^	*p*-Value
	High Risk	Low Risk				
	*n* (%)	*n* (%)				
**Impaired**	47 (74.6)	16 (25.4)	63 (100)	2.63–9.60	26.13	<0.001
**Normal**	62 (36.9)	106 (63.1)	168 (100)			

## Data Availability

Not applicable.

## Supplementary Materials

The following supporting information can be downloaded at: https://www.mdpi.com/article/10.3390/ijerph192316015/s1. Supplementary Material Table S1: STROBE Statement—checklist of items that should be included in reports of observational studies.
