# Supplementary material for: Tooth Loss, Cognitive Impairment and Fall Risk: A Cross-Sectional Study of Older Adults in Rural Thailand
Source: Int J Environ Res Public Health. 2022 Nov 30;19(23):16015. doi: 10.3390/ijerph192316015 (PMC9735973; doi:10.3390/ijerph192316015)
Supplement: Supplementary file 1 [file ijerph-19-16015-s001.zip › ijerph-1962781-supplementary.pdf]

STROBE Statement—checklist of items that should be included in reports of observational studies

	Item No.	Recommendation	Page No.	Relevant text from manuscript
**Title and abstract**	1	(*a*) Indicate the study's design with a commonly used term in the title or the abstract	1	Risk Factors for Fall in Rural-Dwelling Older Adults with Tooth Loss-Induced Cognitive Impairment: A Cross-Sectional Study
		(*b*) Provide in the abstract an informative and balanced summary of what was done and what was found	1	This cross-sectional study investigated socioeconomic and dental factors affecting cognitive func-tions, and the association between tooth loss, cognitive functioning, and fall risk.
**Introduction**				
Background/rationale	2	Explain the scientific background and rationale for the investigation being reported	1-2	Poor oral health, cognitive impairment, and fall risk are interrelated in complex ways.<br>………….. Periodontitis is the major cause of tooth loss and affects masticatory functions and quality of life. Additionally, poor oral health and periodontitis are associated with several extra-oral diseases such as diabetes mellitus, cardiovascular disease, rheumatoid arthritis, and ocular disease [8].
Objectives	3	State specific objectives, including any prespecified hypotheses		With the foregoing in mind, this present study aimed to investigate how far socioeco-nomic and dental factors were associated with cognitive functioning, as well as the association between cognitive functioning and fall risk in older adults living in rural areas. We hypothesized that the combined effects of socioeconomic and dental factors predicted cognitive functioning in this population group.
**Methods**				
Study design	4	Present key elements of study design early in the paper	2	The participants in this cross-sectional study were purposively selected from pa-tients treated at the Dental Service Unit in Chik Thoeng subdistrict, Tan Sum district, Ubon Ratchathani province, Thailand.
Setting	5	Describe the setting, locations, and relevant dates, including periods of recruitment, exposure, follow-up, and data collection	2-3	The participants in this cross-sectional study were purposively selected from patients treated at the Dental Service Unit in Chik Thoeng subdistrict, Tan Sum district, Ubon Ratchathani province, Thailand. Four hundred thirty-one rural-dwelling adults aged from 60 to 74 years were screened to see if they met the inclusion criteria.<br>………………… In line with World Health Organization criteria, the Decayed, Missing and Filled Tooth (DMFT) Index was employed and a detection record form

				was used to record the number of decayed, missing, and filled teeth [13].
Participants	6	*Cross-sectional study*—Give the eligibility criteria, and the sources and methods of selection of participants	2, 3	The inclusion criteria were being residents of Chik Thoeng subdistrict, aged ≥ 60, being able to read and write in Thai, and being consent to participate in the project. It had been decided to exclude patients with dementia because of the likely inability to complete questionnaires and interviews.<br><br>The stratified random sampling method was used to obtain 231 participants.
Variables	7	Clearly define all outcomes, exposures, predictors, potential confounders, and effect modifiers. Give diagnostic criteria, if applicable		Socioeconomic data (age, sex, education levels, family status, congenital disease, body mass index [BMI], activities of daily living [ADLs], and betel chewing) were obtained from a questionnaire, which comprised both closed and open-ended questions. The ADLs were evaluated using Barthel's ADL index with a Cronbach's alpha reliability coefficient of 0.90 [11]. The evaluation form had ten items on the ADL checklist on bathing, dressing, bowel and bladder care, feeding, grooming, climbing stairs, ambula-tion, transfer, and toilet use. The summed ADL scores ranged from 0-20 and were di-vided into two groups – dependent (<12) and independent (≥12).<br>Data on health status (chronic disease, blood pressure, blood glucose levels, et cetera.) were directly obtained from face-to-face interviews and supplemented by rec-ords data from the Java Health Center Information System (JHCIS), Ministry of Public Health. Where there were discrepancies, information was completed after consulting local district hospital staff. Fall risk was measured using the Morse Fall Scale (MFS). The MFS consists of 6 items: history of falling (scored no = 0, yes = 35), ambulatory aid (bed rest/nurse assist = 0, cane/crutches/walker = 15, special furniture/appliances = 30), secondary diagnosis (no = 0, yes =15), gait (normal/ immobile/bed rest = 0, weak = 10, impaired = 20), intravenous (IV) or heparin lock (no = 0, yes = 20), and mental status (oriented to own ability = 0, forgets limitations = 15). In the present study, the partici-pants with MFS scores < 45 and ≥ 45 were classified as low risk and high risk of fall, respectively. Oral examination of respondents was performed by a dentist. The Com-munity Periodontal Index of Treatment Needs (CPITN) form was used to assess perio-dontal status [12]. In line with World Health Organization criteria, the Decayed, Missing and Filled Tooth (DMFT) Index was employed and a detection record form was used to record the

				number of decayed, missing, and filled teeth [13]. Since Luo and co-workers reported that missing > 16 teeth was associated with cognitive impairment, we set this cutoff point for our data analysis [3].
Data sources/ measurement	8*	For each variable of interest, give sources of data and details of methods of assessment (measurement). Describe comparability of assessment methods if there is more than one group	3	The stratified random sampling method was used to obtain 231 participants. So-cioeconomic data (age, sex, education levels, family status, congenital disease, body mass index [BMI], activities of daily living [ADLs], and betel chewing) were obtained from a questionnaire, which comprised both closed and open-ended questions. The ADLs were evaluated using Barthel's ADL index with a Cronbach's alpha reliability coefficient of 0.90 [11]. The evaluation form had ten items on the ADL checklist on bathing, dressing, bowel and bladder care, feeding, grooming, climbing stairs, ambula-tion, transfer, and toilet use. The summed ADL scores ranged from 0-20 and were di-vided into two groups – dependent (<12) and independent (≥12).<br>Data on health status (chronic disease, blood pressure, blood glucose levels, et cetera.) were directly obtained from face-to-face interviews and supplemented by rec-ords data from the Java Health Center Information System (JHCIS), Ministry of Public Health. Where there were discrepancies, information was completed after consulting local district hospital staff. Fall risk was measured using the Morse Fall Scale (MFS). The MFS consists of 6 items: history of falling (scored no = 0, yes = 35), ambulatory aid (bed rest/nurse assist = 0, cane/crutches/walker = 15, special furniture/appliances = 30), secondary diagnosis (no = 0, yes =15), gait (normal/ immobile/bed rest = 0, weak = 10, impaired = 20), intravenous (IV) or heparin lock (no = 0, yes = 20), and mental status (oriented to own ability = 0, forgets limitations = 15). In the present study, the partici-pants with MFS scores < 45 and ≥ 45 were classified as low risk and high risk of fall, respectively. Oral examination of respondents was performed by a dentist. The Com-munity Periodontal Index of Treatment Needs (CPITN) form was used to assess perio-dontal status [12]. In line with World Health Organization criteria, the Decayed, Missing and Filled Tooth (DMFT) Index was employed and a detection record form was used to record the number of decayed, missing, and filled teeth [13].
Bias	9	Describe any efforts to address potential sources of bias	-	None

Study size	10	Explain how the study size was arrived at	3	The stratified random sampling method was used to obtain 231 participants from 431 rural-dwelling adults.
Quantitative variables	11	Explain how quantitative variables were handled in the analyses. If applicable, describe which groupings were chosen and why	3	The Chi-square (χ^2) test and Fisher's exact test were used to investigate the relation-ship between categorical variables. Binary logistic regression analysis was used to an-alyze predictive values of socioeconomic characteristics that might be associated with cognitive impairment. Prerequisites for binary logistic regression were checked for multicollinearity amongst independent variables and independence between observations. The method of binary logistic regression was stepwise. The independent variables were age [< 70 vs ≥ 70 years old], ADLs [< 12 versus ≥ 12] [14], and the number of teeth lost [≤ 16 vs > 16] [3], and were analyzed using predicted binary variables (normal vs impaired cognitive function).
Statistical methods	12	(*a*) Describe all statistical methods, including those used to control for confounding	3	2.2. Statistical analysis<br>The data were analyzed using the Statistical Package for Social Sciences (SPSS version 18, SPSS, Inc., Chicago, IL, USA). Descriptive statistics were assembled using average values, standard deviation (SD), and percentage distribution. The Chi-square (χ^2) test and Fisher's exact test were used to investigate the relationship between cate-gorical variables. Binary logistic regression analysis was used to analyze predictive values of socioeconomic characteristics that might be associated with cognitive im-pairment. Prerequisites for binary logistic regression were checked for multicollinearity amongst independent variables and independence between observations. The method of binary logistic regression was stepwise. The independent variables were age [< 70 vs ≥ 70 years old], ADLs [< 12 versus ≥ 12] [14], and the number of teeth lost [≤ 16 vs > 16] [3], and were analyzed using predicted binary variables (normal vs impaired cognitive function). The results from the binary logistic regression are expressed as odds ratios. Odds ratios > 1 indicate that the event is more likely to occur as the value of the predictor increases. On the other hand, odds ratios < 1 indicate that the event is less likely to occur as the predictor value increases. The sta-tistical significance level was set at P < .05.
		(*b*) Describe any methods used to examine subgroups and interactions	-	Not relevant.
		(*c*) Explain how missing data were addressed	-	Not applicable.

		(*d*) *Cross-sectional study*—If applicable, describe analytical methods taking account of sampling strategy	3	The stratified random sampling method was used to obtain 231 participants from 431 rural-dwelling adults.
		(*e*) Describe any sensitivity analyses		None.
**Results**				
Participants	13*	(a) Report numbers of individuals at each stage of study—eg numbers potentially eligible, examined for eligibility, confirmed eligible, included in the study, completing follow-up, and analysed	3	Four hundred thirty-one rural-dwelling adults aged from 60 to 74 years were screened to see if they met the inclusion criteria.<br><br>The stratified random sampling method was used to obtain 231 participants from 431 rural-dwelling adults.
		(b) Give reasons for non-participation at each stage	3	The sampling process was terminated after reaching the required number of participants.
		(c) Consider use of a flow diagram	-	Not applicable.
Descriptive data	14*	(a) Give characteristics of study participants (eg demographic, clinical, social) and information on exposures and potential confounders	3	The participants in this cross-sectional study were purposively selected from pa-tients treated at the Dental Service Unit in Chik Thoeng subdistrict, Tan Sum district, Ubon Ratchathani province, Thailand. Four hundred thirty-one rural-dwelling adults aged from 60 to 74 years were screened to see if they met the inclusion criteria. The in-clusion criteria were being residents of Chik Thoeng subdistrict, aged ≥ 60, being able to read and write in Thai, and being consent to participate in the project. It had been decided to exclude patients with dementia because of the likely inability to complete questionnaires and interviews.
		(b) Indicate number of participants with missing data for each variable of interest	3	The stratified random sampling method was used to obtain 231 participants from 431 rural-dwelling adults. The sampling process was terminated after reaching the required number of participants.
		Cross-sectional study—Report numbers of outcome events or summary measures	3	The thirteen general and dental characteristics of 231 participants are shown in Table 1.
Main results	16	(*a*) Give unadjusted estimates and, if applicable, confounder-adjusted estimates and their precision (eg, 95% confidence interval). Make clear which confounders were adjusted for and why they were included	5	See Table 2. Factors associated with level of cognitive functioning among participants.

Other analyses	17	Report other analyses done—eg analyses of subgroups and interactions, and sensitivity analyses	-	None.
**Discussion**				
Key results	18	Summarise key results with reference to study objectives		This study shows that tooth loss is associated with cognitive impairment and cog-nitive impairment is related to fall risk in older rural-dwelling persons. Our conclusion is based on the following findings: 1) tooth loss is a predicting factor for cognitive im-pairment and 2) cognitive impairment is associated with fall risk.<br>The relationship between tooth loss and these two variables is complex and bidi-rectional. In Thailand a large number of older adults are affected by high rates of tooth loss [14]. To date, the links between tooth loss, cognitive impairment, and fall risk have been separately reported for different population groups, but more evidence is needed before the overall picture becomes clear. The more obvious direction of causality is that adults with cognitive impairment and increased fall risk are more likely to lose teeth because of restricted ADLs and poor self-care [1a,16]. However, as noted earlier, peri-odontal disease has been linked to changes in the cerebrum, cerebral cortex and white matter functions [4, 15]. Akashi and colleagues' narrative review suggests that odon-togenic infection can lead to intracranial infection, particularly temporal lobe infection [16]. Yang and associates report that bacteria diffused from odontogenic foci to fron-toparietal areas [17], areas responsible for attention control [18]. The medial area of the temporal lobe controls memory function as evidenced by medial temporal lobe at-rophy in elderly individuals with memory impairment [19]. Another possible mecha-nism affecting brain function is bacteriological infection via neuronal pathways, such as the cranial nerve [20].<br>Plozer and colleagues suggest that investigation of pathways by which tooth loss-related morbidity and mortality is mediated will increase the perceived value of dental treatment for general health [21]. Tooth loss without use of dentures is said to be an independent predictor of incident falls in older adults without cognitive im-pairment [22]. These authors propose that people with tooth loss are susceptible to de-pression, which is a risk factor for falls [23]. In many cases depression is associated with cognitive problems [24], which makes it difficult to isolate tooth loss from cogni-tive impairment, supporting our finding of an association between

				cognitive impairment and high risk of falls in older persons.<br>Access to dental treatment is likely to become more difficult for rural populations in a period of economic turbulence and constrained public resources, especially in de-veloping countries such as Thailand [25]. Our participants were older rural-dwellers who mostly live at a distance from medical services. Most only received basic dental care such as scaling and tooth extraction due to the limited coverage provided by the UCS [7]. We have provided further evidence of the association between tooth loss, cognitive impairment and fall risk. If the relationships are indeed bidirectional then tooth loss has an impact on the older rural population that goes far beyond matters like appearance and eating to affect general health status and social and cognitive func-tioning. Service planners need to take account of these wide-ranging impacts to give higher priority to dental services as a determinate of general health status, and make corresponding improvements to patterns of service delivery.
Limitations	19	Discuss limitations of the study, taking into account sources of potential bias or imprecision. Discuss both direction and magnitude of any potential bias	6	The nature of the cross-sectional study limited the outcomes of this study – no temporal link between the outcomes and the exposure was obtained. Another limita-tion was a lack of data on physical strength determinants such as lower limb muscle power and postural balance, which are preventive factors of falls in older adult indi-viduals [26].
Interpretation	20	Give a cautious overall interpretation of results considering objectives, limitations, multiplicity of analyses, results from similar studies, and other relevant evidence	6	These factors might also be related to age-related muscle degeneration and a decline in physical activity [27]. Tooth loss-induced malnutrition and sequential muscle protein wasting should also be considered for further study [28].
Generalisability	21	Discuss the generalisability (external validity) of the study results	6	In addition, our findings were obtained from a small group of older adults; thus, the generalizability of the results must be cautiously performed in other populations.
**Other information**				
Funding	22	Give the source of funding and the role of the funders for the present study and, if applicable, for the original study on which the present article is based	6	This research project was financially supported by Mahasarakham University.

*Give information separately for cases and controls in case-control studies and, if applicable, for exposed and unexposed groups in cohort and cross-sectional studies.
